# Mannan Enhances IL-12 Production by Increasing Bacterial Uptake and Endosomal Degradation in *L. acidophilus* and *S. aureus* Stimulated Dendritic Cells

**DOI:** 10.3389/fimmu.2019.02646

**Published:** 2019-11-15

**Authors:** Ronja Mathiesen, Helene M. S. Eld, Juliane Sørensen, Eva Fuglsang, Lisbeth Drozd Lund, Valentina Taverniti, Hanne Frøkiær

**Affiliations:** ^1^Department of Veterinary and Animal Sciences, University of Copenhagen, Copenhagen, Denmark; ^2^Department of Food, Environmental and Nutritional Science, Università degli Studi di Milano, Milan, Italy

**Keywords:** dendritic cells, Gram-positive bacteria, mannose receptor, IL-12, endosomal killing, phagocytosis, SLAMF1

## Abstract

The mannose receptor (MR) is a C-type lectin involved in endocytosis and with a poorly defined ability to modulate cellular activation. We investigated the effect of mannan treatment prior to stimulation of murine bone marrow-derived dendritic cells with the Gram-positive bacteria *Lactobacillus acidophilus* NCFM *(L. acidophilus*) on the induction of Interleukin (IL)-12. Mannan enhanced the IL-12 production induced by *L. acidophilus* in a dose dependent manner (up to 230% enhancement). Additionally, mannan-enhanced IL-12 induction was also demonstrated with another Gram-positive bacteria, *Staphylococcus aureus (S. aureus)*, while an IL-12 reducing effect was seen on *Escherichia coli* stimulated cells. Furthermore, the expression of Interferon β (*Ifnb*) was increased in cells treated with mannan prior to stimulation with *L. acidophilus*. The addition of mannan but not of bacteria led to endocytosis of MR, while addition of mannan prior to *L. acidophilus* or *S. aureus* resulted in increased endocytosis of bacteria, a faster killing of endocytosed bacteria, and increased reactive oxygen species production. Expression of signaling lymphocytic activation molecule (SLAMF)1 shown previously to be involved in the facilitation of endosomal degradation was upregulated by mannan but not by *L. acidophilus* and *S. aureus*. The IL-12 enhancement by mannan but not the IL-12 induced by the bacteria was abrogated by addition of inhibitors of clathrin coated pits (chlorpromazine and monodansylcadaverine). Furthermore, the addition of acid sphingomyelinase, a facilitator of ceramide raft formation, prior to addition of *L. acidophilus* enhanced the IL-12 production and the endocytosis of bacteria. In summary, our results show that mannan increases the IL-12 production induced by some Gram-positive bacteria through MR-endocytosis, which increases bacterial endocytosis and endosomal killing. The differential effect of MR activation on the IL-12 production induced by Gram-positive and Gram-negative bacteria may influence the immune response toward allergens and other glycoproteins.

## Introduction

The mannose receptor (MR) is a C-type lectin (CTL) expressed particularly by macrophages and dendritic cells (1). The roles ascribed to the MR include boosting of antigen presentation and modulation of cellular activation and trafficking. The MR has been reported to function in endocytosis including phagocytosis and micropinocytosis and, like several other CTLs, has been reported to modulate toll like receptor (TLR) signaling (2). TLRs are involved in directing an appropriate cytokine response to the specific microorganism. Several studies from recent years have reported that the compartment from where the engagement of TLR takes place is important for the resulting cytokine production. Kagan and Barton showed that engagement of TLR4 from the endosome induced a stronger IL-12 response as when engagement of TLR4 happens from the plasma membrane, a phenomenon called compartmentalization (3). Likewise, we and others have shown that endosomal engagement of TLR2 initiates a distinct signaling pathway, resulting in stronger IL-12 induction than if TLR2 stimulation takes place from the plasma membrane (4–6). The increased IL-12 production upon endosomal TLR-ligation is caused by the induction of IFN-β, which in turn induces IL-12 production (7, 8). Accordingly, endocytosis and endocytic receptors play indirectly a key role in both the recognition and the type of response toward bacteria.

A broad diversity of Gram-positive bacteria have been shown to induce a strong IL-12 production but only upon endocytosis (5, 9, 10), which is in full accordance with the compartmentalization principle. Very often, carbohydrates on the bacterium seem to be involved in the endocytosis, and this makes the CTLs obvious receptor candidates involved in the endocytic event. The CTL family comprises several receptors recognizing various carbohydrates often found on microorganisms, e.g., 1,4 β-glucans and mannan (11). The receptor dectin-1 recognizing β-glucan is involved in endocytosis of yeast (12), while the MR has been shown to recognize mannan and other mannose-containing carbohydrate structures present on various bacteria and viruses (13, 14) and to collaborate with TLR2 in inducing cytokine production (2). To date, a direct role of the MR as a phagocytotic receptor has however not been established. Rather, MR-facilitated endocytosis of mannan and mannose-containing glycoproteins takes place through clathrin coated pits (13). In contrast to dectin-1 and most other CTLs, the MR does not contain an intracellular signaling motif and is thus not capable of inducing a cytokine response on its own. However, the MR contains an internalization motif, and reports of a cytokine modulating activity of MR do exist (1). MR engagement and endocytosis has been shown to reduce LPS-induced IL-12 in dendritic cells (15) and to increase TLR2-mediated activation of MAP kinases in HEK 293 cells co-expressing MR (2). The effect of MR ligation on the cytokine response toward Gram-positive bacteria has not been investigated. As Gram-positive bacteria employ different receptors and mechanisms than Gram-negative bacteria and LPS (15), it is likely that the effect of mannan is also different.

*Lactobacillus* strains induce IL-12 production to a widely varying degree (16). We have shown that a high IL-12 induction depends on endocytosis of the bacteria, which leads to endosomal degradation and the induction of IFN-β (4, 7). *L. acidophilus* is among the strains inducing the highest production of IL-12 (9, 16, 17). Also *S. aureus*-induced IL-12 is dependent on endocytosis (5), while Gram-negative bacteria such as *E. coli* often give rise to IL-12 without prior endocytosis, although endocytosis and TRIF mediated signaling may lead to a higher IL-12 production (17, 18).

To assess the involvement of the MR on the IL-12 production induced by *Lactobacillus* and other Gram-positive bacteria, we used murine bone marrow derived dendritic cells (BMDCs). We added mannan to BMDCs prior to stimulation with bacteria in order to characterize the effect of mannan on the IL-12 induction.

## Materials and Methods

### Bacterial Strains

*Lactobacillus acidophilus* NCFM (*L. acidophilus*) (Dupont, Finland) and *Escherichia coli* Nissle 1917 O6:K5:H1 (*E. coli*) (Statens Serum Institut, Copenhagen, Denmark) were grown from frozen stocks (−80°C). *L. acidophilus* were grown anaerobically overnight (o/n) at 37°C on de Man Rogosa Sharp (MRS) broth (Merck, Darmstadt, Germany), while *E. coli* were grown aerobically o/n at 37°C on Luria-Bertani (LB) broth (Merck). The laboratory *Staphylococcus aureus* strain NCTC8325-4 (19) was grown aerobically o/n at 37°C on tryptic soy agar (TSA) and inoculated in tryptic soy broth (TSB; Difco) to reach stationary phase (OD_600_ >600). Subsequently, 0.5% of the o/n culture was inoculated into fresh TSB and grown to exponential phase (OD_600_ <1). For stimulation, a multiplicity of infection (MOI) of 2 for *L. acidophilus*, 10 for *E. coli* and 12 for *S. aureus*, corresponding to the MOI giving rise to the highest IL-12 production, was used.

### Generation of Murine Dendritic Cells

BMDCs were prepared from 6 to 12 week old mice (C57BL/6, Taconic, Lille Skensved, Denmark) by the protocol of Lutz et al. (20), with slight modifications as previously described (21). Cells were cultivated in RPMI 1640 with 10% heat-inactivated fetal calf serum in the presence of granulocyte-macrophage colony-stimulating factor (GM-CSF).

### Ligands and Inhibitors

Mannan from the yeast *Saccharomyces cerevisiae* and laminarin from the brown seaweed *Laminaria Digitata* (both from Sigma Aldrich, St. Louis, MO, USA) were used in the concentrations indicated in each experiment. Anti-IFN-β antibody (clone AF 585-NA, R&D Systems, Minneapolis, MN USA) was used in the concentration 10 μg/ml. Cytochalasin D (CytD), Chlorpromazine (CPZ), acid sphingomyelinase (ASMase), monodansylcadaverine (MDC), and Nystatin (all from Sigma- Aldrich) were used in a final concentration of 0.5 μg/mL, 10 μM, 0.1 U/mL, 10, and 10 μM, respectively.

### Cell Surface Expression of the MR and Signaling Lymphocytic Activation Molecule (SLAMF)1

Immature BMDCs (2 · 10^6^ cells/mL) were re-suspended in fresh medium without GM-CSF, seeded in 96-well-tissue cultures plates (150 μl/well) (Nunc, Roskilde, Denmark), and incubated with mannan, laminarin, *L. acidophilus, E. coli*, or *S. aureus* for 30 min. After incubation, the cells were incubated with anti-mouse FcγRII/III (BD Biosciences, San Jose, CA) for 10 min, incubated with PE-conjugated anti-mouse MR/CD206 (clone FAB2535p) or PE-Cy7–conjugated anti-mouse SLAMF1/CD150 (clone mShad150) (R&D Systems, Minneapolis, MN USA) for 45 min on ice, and then washed twice in Dulbecco's Phosphate-Buffered Saline (DPBS) containing 1% FCS and fixed in 1% formaldehyde. The samples were analyzed on a BD FACS Canto II flow cytometer (BD Biosciences, San Jose, CA) based on counting 10,000 cells. Dead cells were excluded based on their forward and side scatter characteristics. Data analysis was performed using the software program Flowjo (Treestar, Ashland, OR).

### Endocytosis Assay and ROS Production

BMDCs (2 · 10^6^ cells/mL) re-suspended in fresh medium without GM-CSF were seeded in 96-well-tissue cultures plates (150 μL/well) and incubated with or without CytD for 60 min prior to addition of mannan (100 μg/mL). The cells were then incubated for 60 min with Alexa Fluor (AF) 647-labeled *L. acidophilus or S. aureus* in MOI 2 and 12, and for 10 min with fluorescein isothiocyanate (FITC)-conjugated dextran (150 kDa, Sigma Aldrich, St. Louis, MO, USA). Cells were washed twice in DPBS containing 1 % FCS and fixed in 1% formaldehyde. All incubation steps were performed at 37°C in 5% CO_2._ The uptake of the AF647-labeled bacteria or FITC-conjugated dextran was analyzed with the BD FACSCanto II flow cytometer (BD Biosciences, San Jose, CA). Data analysis was performed on live single cells using the software program Flowjo (Treestar, Ashland, OR). Reactive oxygen species (ROS) production was assessed by incubating BMDCs with 5 μM redox-sensitive probe, 5-(and 6-) chloromethyl-2′-7′-dichlorodihydrofluorescein diacetate, acetyl ester (CM-H_2_DCFDA) (Thermo Fisher). Oxidation was detected by the increase in fluorescein (FITC) intensity by flow cytometry and stimulated samples were compared to non-stimulated and lipopolysaccharide (LPS) stimulated samples and to samples without CM-H_2_DCFDA added.

### Cytokine Quantification and Expression

BMDCs (2 · 10^6^ cells/mL) re-suspended in fresh medium without GM-CSF were seeded into 48-well-tissue cultures plates (500 μL/well) (Nunc, Roskilde, Denmark). The cells were pretreated with mannan or laminarin for 30 min prior to stimulation with bacteria for 20 h. The concentration of IL-12(p70) and IL-10 in the culture supernatants was measured after the 20 h stimulation with bacteria using Duoset ELISAs (R&D Systems, Minneapolis, MN, USA). The concentration of IFN-β was determined by a commercially available ELISA kit (PBL Assay Science, Piscataway, NJ) according to the manufacturer's instructions.

### Quantitative Real Time PCR Analysis

For the determination of gene expression, cells were pretreated with mannan for 30 min prior to stimulation with bacteria for 2, 4, 6, and 10 h. Total RNA was extracted by MagMAX Express (Applied Biosystem, Foster City, CA) using the MagMAX-96 RNA Isolation Kit (Ambion, Austin, TX) following the supplier's protocol, including a DNAse treatment for genomic DNA removal. RNA quality was verified by Bioanalyzer (Agilent, Santa Clara, USA) and the concentration was determined by Nanodrop (Thermo, Wilmington, USA).

Total RNA (500 ng) was reverse transcribed by the High-Capacity cDNA Reverse Transcriptase Kit (Applied Biosystems, Foster City, CA), using random hexamer primers according to the manufacturers' instructions. The expression of the genes encoding IFN-β and β-actin was detected using primers and probes, as previously described [4].

For each sample, 2 μL cDNA (3 ng/μL) was amplified in triplicates on a StepOnePlus by using universal fast thermal cycling parameters and TaqMan Fast universal PCR Mastermix (both from Applied Biosystems, Foster City, CA) in a total reaction volume of 10 μL. Fold changes in gene expression were calculated by the comparative cycle threshold (CT) method (22). The expression of target genes was normalized to β-actin as reference gene [ΔCT = CT(target) – CT(reference)]. Fold change in gene expression was calculated as 2^−ΔΔCT^ where ΔΔCT = ΔCT(sample) – ΔCT(calibrator), and where the average ΔCT of samples from controls at 0 h of stimulation was used as calibrator.

### Western Blotting

Cell lysates were prepared by re-suspending BMDCs (1.1 · 10^7^ cells/mL) in fresh media and incubated with or without mannan for 1 h. Cells were spun down for 5 min (241 g, 4°C) and the cell culture supernatants were collected, and protease inhibitors (1X concentration, Sigma Aldrich, St. Louis, MO, USA), 10 mM EDTA (Sigma Aldrich, St. Louis, MO, USA), and 1 mg/mL BSA were added and stored at −80°C until analysis. After washing of the cells with 150 μL/well of ice-cold DPBS, the cells were lysed with 100 μL/well of RIPA buffer (Thermo Scientific/Pierce) supplemented with protease inhibitor cocktail and 2 mM EDTA (Sigma-Aldrich). The mixture was kept on ice for 30 min with occasional swirling using a vortex mixer. The cells were spun down (20 min. at 18,000 g, 4°C), and the supernatant and cells were collected and stored at −80°C until analysis. All samples were mixed with sample buffer (Invitrogen), boiled for 5 min, resolved under non-reducing conditions on a NuPAGE 7% Tris-Acetate gel (Invitrogen) and transferred to nitrocellulose membrane (Invitrogen) using the iBlot™ dry-blotting. After blocking for 90 min with 5% skimmed milk in 10 mM Tris buffer 0.5% Tween 20, the membrane was incubated for 24 h with rat-anti-MR antibody (Nordic Biosite) and horseradish peroxidase-conjugated polyclonal rabbit anti-rat immunoglobulin (Dako, Denmark). The bound antibodies were detected using ECL reagents according to the manufacturer's instructions (GE Healthcare, Denmark).

### Survival of Endosomal Bacteria

Overnight cultures of *L. acidophilus* and *S. aureus* were centrifuged, the pellet was washed with sterile PBS, and bacterial suspensions (1 × 10^9^ cells/mL) were prepared. Aliquots of the bacterial suspensions were used to prepare serial dilution and plating on MRS agar or TSA, as a reference for the viability status of the bacteria. Bacteria were added to 2 × 10^6^ BMDCs (MOI 50) in 6-well-plates with or without prior addition of 100 μg/ml mannan. After 1 h of incubation, internalization of bacteria by BMDCs was blocked by washing the cells with cold PBS twice, while keeping plates on an ice tray. Media containing 100 μg/ml of gentamycin (Sigma-Aldrich) was then added to BMDCs, and plates were incubated for 1 h to allow the killing of remaining non-endocytosed bacteria. The number of endosomal bacteria was evaluated by collecting BMDCs at this point (0 h) or 1 and 2 h after the gentamycin treatment (corresponding to 2, 3, or 4 h after bacterial stimulation). At each time point, BMDCs were harvested and resuspended in 0.1% Triton X-100 in DPBS and agitated for 30 min at room temperature. The number of colony forming units (CFU) per ml was determined by counting plated serial dilutions upon incubation for 72 h at 37°C in jars for anaerobic conditions (AnaeroGen, Oxoid).

### Statistical Analysis

Data represent mean of measurements from triplicate cultures. Error bars indicate standard deviation. Statistical calculations were performed using the software GraphPad Prism 5 (GraphPad Software, San Diego, CA) by using ANOVA, followed by Dunnets post-test (compared to un-stimulated sample) or Bonferroni multiple comparison test to compare either different groups of multiple data sets, or unpaired Students *t*-test for comparison of two sets of variables. *P*-values of < 0.05 were considered significant and indicated by asterisks (^*^P < 0.05, ^**^P < 0.01, and ^***^P < 0.001).

## Results

### Mannan Increases the IL-12 Induction by *L. acidophilus*

**Figure 1:**
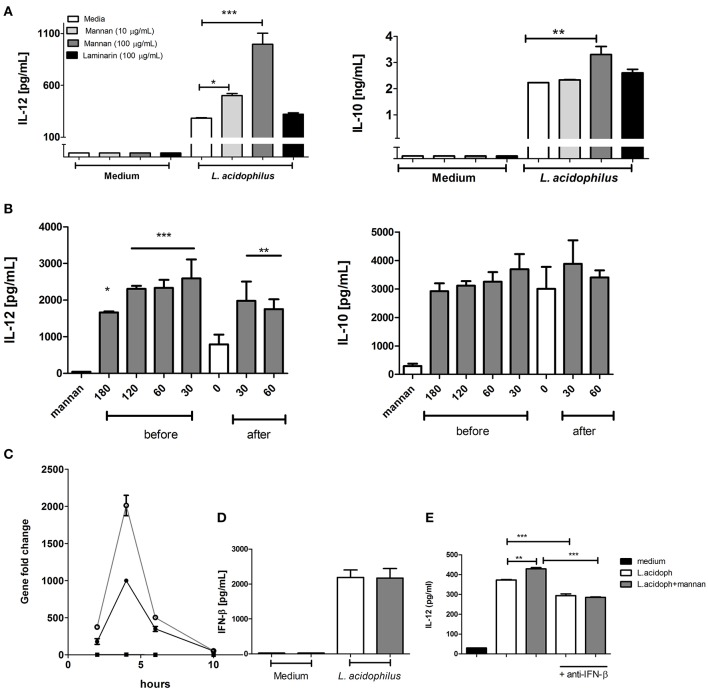
Mannan increases the IL-12 induction by *L. acidophilus*. **(A)** Immature BMDCs were pretreated with the C-type lectin receptor ligands; laminarin at 100 μg/mL or mannan at 10 and 100 μg/mL. After 30 min, the BMDCs were stimulated with *L. acidophilus* NCFM (MOI 2) for 20 h, and the concentration of IL-12 and IL-10 in the supernatant was measured by ELISA. **(B)** Immature BMDCs were pre-treated or post-treated with mannan (100 μg/mL) for 0–180 min prior to or 30–60 min after stimulation with *L. acidophilus* NCFM and subsequently incubated for 20 h. The concentration of IL-12 and IL-10 was measured by ELISA. **(C)** Expression of *Ifnb* was measured by qPCR and normalized to the relative expression of β*-actin* after 2, 4, 6, and 10 h of stimulation. **(D)** IFN-β concentration in supernatants from BMDCs stimulated with *L. acidophilus* with (gray column) or without (white column) 30 min pre-stimulation with mannan. Induction of IFN-β production was measured after 20 h stimulation by ELISA. **(E)** The effect of addition of anti-IFN-β antibody simultaneously with *L. acidophilus* on the production of IL-12 after 20 h of stimulation with or without 30 min mannan pre-treatment. Data are representative of at least two independent experiments and indicate mean ± SD of triplicates (*P < 0.05; **P < 0.01; and ***P < 0.001).

To assess the effect of mannan on IL-12 production by *L. acidophilus* stimulated BMDCs, we treated BMDCs with mannan 30 min prior to stimulation with *L. acidophilus*. As a negative control, we stimulated cells with laminarin, a β-(1-3), (1-6) glucan that is known to bind to dectin-1 (23). Neither mannan nor laminarin induced IL-12 or IL-10 production when added alone (Figure 1A, medium). Mannan significantly increased the IL-12 production induced by *L. acidophilus* in a dose dependent manner (Figure 1A, *L. acidophilus*). Addition of 10 and 100 μg/ml of mannan increased the IL-12 production by ~70 and 230%, respectively. Addition of 10 μg/ml mannan did not affect the IL-10 production, while 100 μg/ml increased the IL-10 production by 30%. The IL-12 enhancing effect was also seen if mannan was added to the cells 1, 2, or 3 h prior to *L. acidophilus* stimulation, although the effect became gradually weaker with the longer pre-incubation times (Figure 1B). Likewise, addition of mannan ½-1 h after stimulation with *L. acidophilus* enhanced the IL-12 production significantly. In contrast, the *L. acidophilus* induced IL-10 production was not significantly increased by mannan at any time points (Figure 1B). This reflects a general pattern of the effect of mannan on *L. acidophilus* induced IL-10; in some experiments we found slight but significant increase while in most experiments, the increase was not significant. We have previously shown that the majority of the *L. acidophilus*-induced IL-12 production is caused by the induction of IFN-β, which through binding to the IFN-*a* receptor (IFNAR) initiates induction of IL-12 (7). Accordingly, we measured the expression of *Ifnb* at 2, 4, 6, and 10 h following *L. acidophilus* stimulation with or without prior addition of mannan (Figure 1C). Mannan alone did not give rise to any expression of *Ifnb. L. acidophilus* induced an increased *Ifnb* expression that peaked at 4 h after stimulation with *L. acidophilus* and decreased to background level at 10 h. Addition of mannan enhanced the expression of *Ifnb*; however, the course of the expression was similar to that without mannan, showing a peak in *Ifnb* expression at 4 h with the expression dropping to background levels after 10 h. Measuring the IFN-β concentration in the supernatant 24 h after stimulation showed no difference between cells with or without addition of mannan (Figure 1D). Moreover, addition of an anti-IFN-β antibody to *L. acidophilus* stimulated cells reduced the IL-12 concentration while abolishing the IL-12 inducing effect of mannan in *L. acidophilus* cells (Figure 1E). This confirms that IFN-β is readily consumed by the cells for induction of IL-12 (7).

### *S. aureus* but Not *E. coli* Induced IL-12 Is Also Enhanced by Mannan

**Figure 2:**
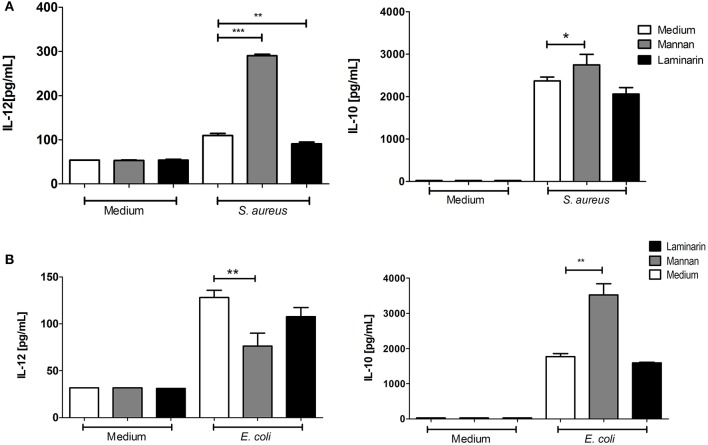
Mannan enhances the IL-12 induction by *S.aureus* but not by *E.coli*. Immature BMDCs were pretreated with laminarin or mannan at 100 μg/mL. After 30 min the BMDCs were stimulated with **(A)** *S. aureus* (MOI 12) or **(B)** *E. coli* Nissle 1917 (MOI 10) for 20 h. Induction of IL-12 and IL-10 was measured by ELISA in culture supernatants. Data are representative of two independent results. Data indicate mean ± SD of triplicates (*P < 0.05; **P < 0.01; and ***P < 0.001).

The Gram-positive *S. aureus*, as well as the Gram-negative *E. coli*, induce IL-12 production in BMDCs (4, 5). Addition of mannan or laminarin alone had no effect on the IL-12 and IL-10 production (Figure 2, medium). Treating BMDCs with mannan prior to the bacterial stimulation with *S. aureus* enhanced the IL-12 production approximately three-fold and led to a slight increase in IL-10 (Figure 2A). In contrast, addition of mannan prior to stimulation with *E. coli* led to a reduced IL-12 production and a two-fold upregulation of IL-10 (Figure 2B). Hence, only in BMDCs stimulated with the Gram-positive bacteria *L. acidophilus* and *S. aureus* did mannan increase the production of IL-12.

### Mannan Reduces the MR Surface Expression

**Figure 3:**
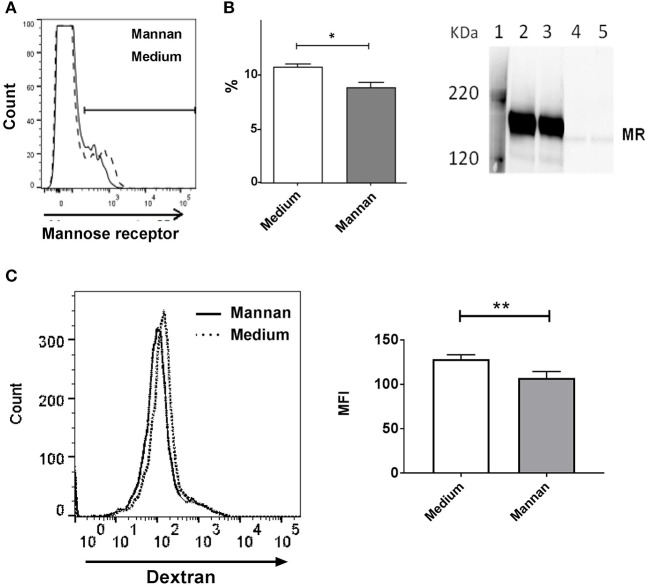
Addition of mannan decreases expression of MR on BMDCs. **(A)** Cell surface expression of MR measured by flow cytometry in BMDCs treated with medium (dashed line) or mannan (100 μg/mL, solid line) for 30 min prior to staining with PE-labeled anti-MR antibody. The percentages of MR positive cells (%) are depicted in the histogram. **(B)** Immune blotting: BMDCs were added with medium (lane 2 and 4) or mannan (100 μg/mL, lane 3 and 5), and after 1 h supernatants (lane 4 and 5) and lysed cells (lane 2 and 3) were analyzed for the content of MR by Western blotting. **(C)** FITC positive cells in BMDC pretreated with medium (dotted line) or mannan (black solid line) and then added with FITC-dextran were measured by flow cytometry. The mean fluorescence intensities (MFI) are depicted in the histogram. Data indicate mean ± SD of duplicates (*P < 0.05 and **P < 0.01).

Addition of mannan to the BMDCs resulted in a decreased surface expression of the mannose receptor (Figure 3A). Of note, only a minority (~10%) of the cells expressed MR, which is in accordance with our earlier studies, as well as other studies showing that only around 10% of BMDCs are fully differentiated (7, 18).

Immunoblotting showed that untreated cells expressed an amount of MR comparable to cells treated with mannan (Figure 3B, lane 2 and 3, respectively), and almost no MR was present in the cell supernatant of untreated and treated cells (Figure 3B, lane 4 and 5, respectively), indicating minimal shedding. Testing the MR expression at different time points after addition of mannan showed that the MR expression was reduced after 30 min where after it returned to the level prior to addition of mannan (data not shown). Endocytosis of FITC-conjugated dextran, which has been demonstrated to bind to and become internalized by the MR (24), was inhibited by mannan (Figure 3C), thus confirming that endocytosis through the MR is a major route of mannan uptake in dendritic cells.

### Mannan Enhances Endocytosis and Endosomal Killing of *L. acidophilus*

**Figure 4:**
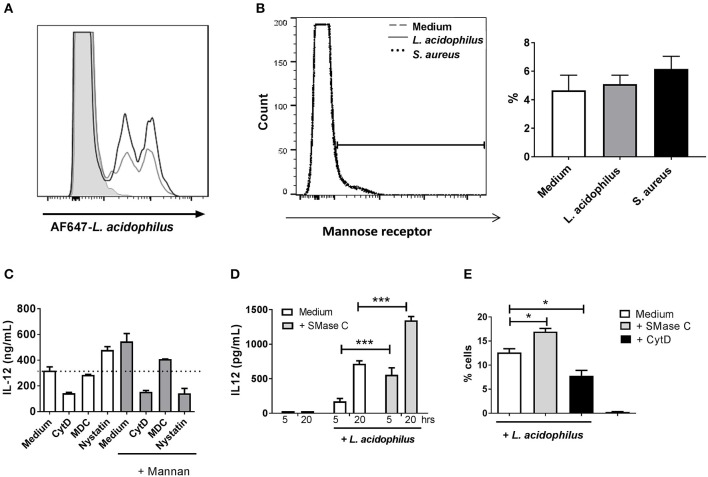
Mannan increases endocytosis of *L. acidophilus* but does not act as a phagocytic receptor. **(A)** Mannan (100 μg/ml) (black line) or medium (gray line) was added to immature BMDCs 30 min before addition of AF647-labeled *L. acidophilus* (MOI 50). The number of cells that had endocytosed bacteria after 1 h of incubation was assessed by flow cytometry. **(B)** Cell surface expression of the MR measured by flow cytometry in BMDCs treated with medium (dashed line), *L. acidophilus (*MOI 2, solid line) or *S. aureus* (MOI 12, dotted line) for 30 min prior to staining the MR with PE-labeled anti-MR antibody. The percentages of MR positive cells (%) were obtained from the gate set in the histogram. **(C)** IL-12 production in BMDCs pretreated with medium, monodansylcadaverine (MDC), nystatin, or cytochalasin D (CytD) for 1 h and with media (white columns) or mannan (gray columns) for 30 min and then stimulated with *L. acidophilus* for 20 h. IL-12 concentration in supernatant was measured by ELISA. **(D)** Cells were added ASMase (0.1 U/ml) for 1 h, then stimulated with *L. acidophilus* and incubated for 5 or 20 h. IL-12 concentration in supernatant was measured by ELISA. **(E)** The proportion of *L. acidophilus* positive cells after pre-treatment of BMDCs with ASMase or CytD for 30 min prior to incubation with *L. acidophilus* for 1 h. *P <0.05; ***P < 0.001.

To investigate whether addition of mannan prior to bacterial stimulation increases the uptake of bacteria by the BMDCs, we added fluorescence-labeled bacteria to the BMDCs 30 min after addition of mannan and measured the number of positive cells by flow cytometry. Addition of mannan led to increased endocytosis of the AF647-labeled *L. acidophilus* (Figure 4A) and of AF647-labeled *S. aureus* (data not shown). Incubation of BMDCs with either *L. acidophilus* or *S. aureus* did not alter the surface expression of MR (Figure 4B), indicating that the MR is not directly involved as a phagocytic receptor in the endocytosis of the bacteria.

The MR is endocytosed through clathrin coated pits (25). When the inhibitor of clathrin coated pit endocytosis, MDC, was added prior to mannan in *L. acidophilus* stimulated cells, the enhancement in IL-12 production was reduced while MDC did not affect the *L. acidophilus* induced IL-12 (Figure 4C). Ceramide formation is pivotal for phagocytosis, and ceramide formation in rafts displaces cholesterol (26, 27). Treatment of BMDCs with the actin polymerization inhibitor cytochalasin D (CytD) prior to addition of *L. acidophilus* reduced the IL-12 induction regardless if the cells were treated with mannan (Figure 4C). When nystatin, an inhibitor of cholesterol dependent endocytosis and of phagocytosis (28), was added to the BMDCs prior to addition of *L. acidophilus*, the production of IL-12 increased. In contrast, if nystatin was added prior to addition of mannan followed by *L. acidophilus*, the IL-12 production was reduced (Figure 4C), indicating a shift in the mechanism of endocytosis upon mannan pre-stimulation. Ceramide formation is pivotal for phagocytosis and is induced by ASMase. Adding ASMase to the BMDCs prior to stimulation with *L. acidophilus*, led to increased IL-12 production (Figure 4D) and increased uptake of AF647-labeled *L. acidophilus* (Figure 4E), suggesting a role of increased ASMase activity in the increased uptake of *L. acidophilus* after mannan pre-stimulation. Of note, adding ASMase did not affect *E. coli* uptake and IL-12 production (data not shown).

**Figure 5:**
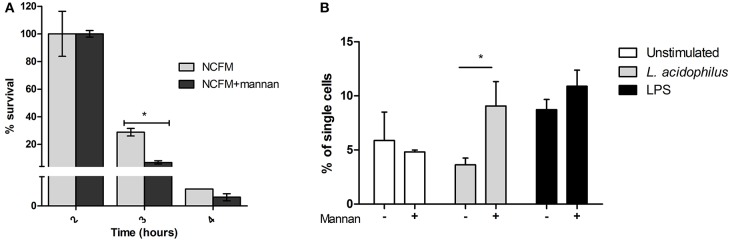
Mannan increases endosomal degradation of *L. acidophilus*. **(A)** *L. acidophilus* (MOI 50) was added to BMDCs and incubated for 1 h. After washing and gentamycin treatment, the number of endocytosed bacteria at 0, 1, or 2 h was counted and the % survival calculated relative to 0 h of incubation. **(B)** ROS production in BMDCs upon stimulation with *L. acidophilus* and LPS with or without mannan for 20 h measured as the oxidation of CM-H2DCFDA. *P < 0.05.

To investigate whether addition of mannan influenced killing of the endocytosed bacteria, which may contribute to the increased IL-12 production, we added live *L. acidophilus* to BMDCs pretreated with or without mannan and assessed the number of CFU after 2, 3, and 4 h of incubation (Figure 5A). The number of live bacteria recovered from the BMDCs decreased dramatically between 2 and 4 h of incubation; in cells without mannan added, the CFU decreased from 3.6·10^5^ to –4.5·10^3^, respectively. Cells pretreated with mannan had after 3 h of incubation only 30% and after 4 h 60% of the bacterial count found in untreated cells. We further assessed whether mannan affected ROS production in *L. acidophilus* stimulated cells and found a doubling of FITC positive cells after addition of mannan prior to stimulation (Figure 5B). LPS used as a positive control, induced ROS production independent of mannan (Figure 5B). Taken together, mannan leads to increased bacterial endocytosis as well as enhanced endosomal killing and ROS production.

### CD150 (SLAMF1) Surface Expression Is Enhanced by Mannan but Not by *L. acidophilus*

**Figure 6:**
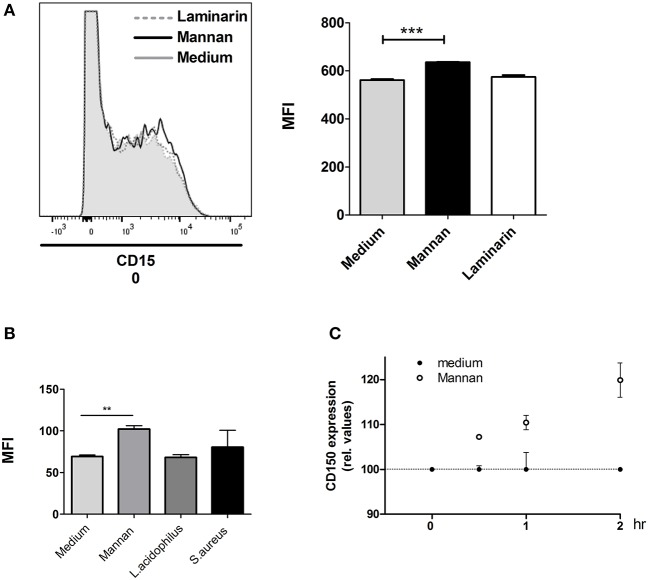
CD150 (SLAMF1) surface expression is enhanced by mannan. **(A)** Immature BMDCs were added with mannan or laminarin and after 30 min, the expression of CD150 was measured by a PE-Cy7 labeled anti-CD150 antibody by flow cytometry. Mean fluorescence intensity (MFI) is depicted to the right. **(B)** Immature BMCDs were added with *L. acidophilus, S. aureus*, or mannan and after 30 min, the expression of CD150 was measured by flow cytometry. Mean fluorescence intensity (MFI) is depicted to the right. **(C)** The expression of CD150 was measured at different time points after addition of mannan (100 μg/ml) to BMDCs. The CD150 expression relative to unstimulated cells at the same time point is shown. **P < 0.01; ***P < 0.001.

MR expression was decreased by mannan and unaltered after bacterial endocytosis, suggesting that the MR is not directly involved as a phagocytic receptor in bacterial endocytosis. As mannan nevertheless affected the endocytosis and bacterial killing as well as IFN-β and IL-12 production in BMDCs stimulated with *L. acidophilus*, endocytosis of MR by mannan may induce activation of other molecules involved in endocytosis and endosomal degradation of microorganisms. Such molecules could be ASMase and CD150 (SLAMF1). SLAMF receptors including CD150 were demonstrated to alter cytokine production induced by phagocytes (29), and CD150 expression was upregulated on the surface of phagocytes together with ASMase upon activation of DC-SIGN in human monocyte derived dendritic cells (30). We speculated that the activation of the MR could lead to a similar activation. Addition of mannan to the BMDCs induced a slight upregulation of CD150 (Figure 6A). Stimulation with *L. acidophilus* or *S. aureus* alone did not lead to increased CD150 expression (Figure 6B). After addition of mannan, we followed the expression of CD150 over time and found that CD150 expression slightly increased after 30 min and increased further during the following 1½ h (Figure 6C).

### Chlorpromazine Abrogates the Mannan Induced IL-12 Enhancement Without Affecting the Enhanced Endocytosis of *L. acidophilus*

**Figure 7:**
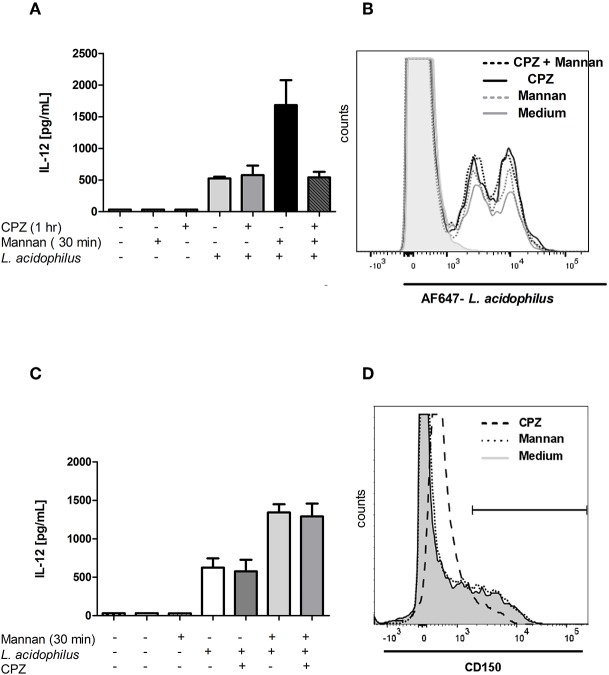
Chlorpromazine abrogates the mannan induced IL-12 enhancement without decreasing endocytosis of *L. acidophilus*. **(A)** Chlorpromazine (CPZ) was added to immature BMDCs 1 h before *L. acidophilus* stimulation. Samples were added with mannan 30 min before *L. acidophilus* stimulation. IL-12 concentration in supernatants harvested after 24 h was measured by ELISA. **(B)** CPZ was added to cells 1 h before and mannan 30 min before addition of AF647-labeled *L. acidophilus*, and after 1 h of incubation with bacteria, the proportion of cells that had endocytosed bacteria was measured by flow cytometry. Solid gray line: *L. acidophilus* + medium, gray dotted: *L. acidophilus* + mannan, black solid: *L. acidophilus* + CPZ, black dotted: *L. acidophilus* + mannan + CPZ. **(C)** Cells were added with mannan 30 min before and CPZ was added simultaneously with *L. acidophilus* to immature BMDCs. IL-12 concentration in supernatants harvested after 24 h was measured by ELISA. **(D)** BMDCs were added with medium, mannan or CPZ 30 min prior to staining the cells with PE-Cy7 labeled anti-CD150 antibodies and assayed by flow cytometry.

CPZ is an inhibitor of endosomal transport (31), and CPZ was shown to inhibit ASMase activity in cells but not before 1 h after of incubation (32). Addition of CPZ 30 min prior to addition of mannan and 1 h prior to stimulation with *L. acidophilus* abrogated the enhanced IL-12 production seen after pre-stimulation with mannan. However, the IL-12 production induced by *L. acidophilus* alone was not affected by CPZ (Figure 7A), indicative of an effect exclusively on MR endocytosis or the MR initiated enhanced signaling. To assess the effect of CPZ on the mannan enhanced uptake of *L. acidophilus*, CPZ was added before addition of mannan and AF647-labeled *L. acidophilus* (Figure 7B). As expected, mannan enhanced the uptake of *L. acidophilus*. Furthermore, CPZ led to an increased bacterial uptake, but mannan together with CPZ did not lead to a further increased uptake. When CPZ was added simultaneously with *L. acidophilus* (i.e., 30 min after mannan addition), the IL-12 enhancing effect of mannan was unaffected (Figure 7C). Addition of CPZ to the BMDCs strongly altered the CD150 expression, resulting in a generally increased CD150 expression 1 h after addition of CPZ (Figure 7D).

## Discussion

The role of the MR in dendritic cells as an antigen sampler is well-established (33) and some data have suggested the MR to be involved in phagosome formation and in the delivery of ligands to early endosomes for sorting and clearance (33, 34); however, the role of the MR is still not fully understood.

Here, we present data that support a role of MR endocytosis in modulating the cytokine response toward bacteria in BMDCs and, notably, that MR endocytosis taking place around the time of the phagocytosis of intact Gram-positive bacteria leads to enhanced production of the Th1-inducing and NK-activating cytokine IL-12. This enhanced production was substantiated by an enhanced endocytosis of the bacteria and faster bacterial degradation and killing in the endosomes of BMDCs. A downregulating effect of MR engagement in TLR4 stimulated dendritic cells on IL-12 production has been demonstrated before (15, 35); however, an enhancing effect on the IL-12 response to endocytosed Gram-positive cells has to the best of our knowledge not been reported before.

We have in BMDCs previously demonstrated that both *S. aureus, L. acidophilus* and other lactobacilli strains induce IFN-β upon endocytosis, which in turn leads to induction of IL-12 (5, 7, 16). Here, we showed that addition of mannan prior to stimulation with *L. acidophilus* or *S. aureus* enhances the induction of IL-12. As expression of *Ifnb* was transiently enhanced in mannan and *L. acidophilus* treated BMDCs, we suggest that mannan enhances the IL-12 production by enhancement of the induction of IFN-β. The expression of *Ifnb* is an early event taking place 2–8 h after stimulation with *L. acidophilus* or *S. aureus* (5, 7). Mannan did not prolong the expression period, merely it enhanced the signal initiated by the bacterial stimulation during this period, in turn resulting in more IL-12. Addition of mannan alone with no microbial stimulation did not induce any cytokine induction, demonstrating that the increase in IL-12 production is not caused by an additive effect. *Ifnb* expression is induced through TLR-ligation and activation within the endosome (4, 5, 36). Thus, increased endocytosis, as well as more efficient degradation of the endocytosed bacteria may lead to enhanced *Ifnb* expression and accordingly, IL-12 induction. Indeed, both the uptake of *L. acidophilus* and its endosomal degradation were enhanced by the mannan pre-stimulation. Of note, IL-10 production was also upregulated by mannan pre- stimulation, albeit not to the same degree as IL-12. IL-10 is known to suppress IL-12 production, but such an effect was apparently not so potent that it could change the IL-12 enhancing effect substantially.

Interestingly, we found that addition of mannan prior to stimulation of BMDCs by *E. coli* inhibited the induced production of IL-12. *E. coli* induces IL-12 production in dendritic cells through TLR4 activating a pathway distinct from the pathway induced by Gram-positive cells (4, 36, 37), which may explain the diverging effects of mannan addition on Gram-positive and Gram-negative bacterial stimulation of BMDCs. To this end, a reduction of the IL-12 production in LPS stimulated human monocyte-derived dendritic cells by mannan was recently reported (15), and mannan showed inhibition of endosomal killing of *E. coli* in macrophages (38). Yurchenko et al. reported that SLAMF1(CD150) is involved in TRIF mediated induction of IFN-β by LPS in macrophages (39). In contrast, *L. acidophilus* employs MyD88 through TLR2 in the induction of IFN-β (7, 36). Whether SLAMF1 is involved in this mechanism remains to be elucidated.

Mannan led to endocytosis of the MR whereby the MR may localize to phagosomes, where it may affect endosome maturation and receptor signaling. This is supported by Heinsbroek et al., who showed that MR from endosomes accumulated in the phagosomal membrane 20 min after phagocytosis of yeast or zymosan (33). MR endocytosis may thus activate other pathways that in turn lead to faster phagosome maturation. Here SLAMF1 may play a role, as we found that SLAMF1 expression was increased upon MR endocytosis. It was previously shown that SLAMF1 is upregulated upon measles virus engagement of DC-SIGN (30), and SLAMF1 has been associated with enhanced activity of the NADPH oxidase NOX2 complex and phagolysosomal maturation (40). Whether SLAMF1 plays a role in the mannan enhanced IL-12 induction by Gram-positive bacteria is however purely speculative; the upregulation of SLAMF1 may also become upregulated in order to prime cells for interaction with and antigen presentation for T cells. Of note, the upregulated SLAMF1 expression lasted for at least 2 h, while the reduction in MR expression was very transient, peaking around 30 min after addition of mannan. This substantiates a role for SLAMF1 as the effect of mannan on IL-12 production was seen when mannan was added up to 3 h prior to stimulation with *L. acidophilus*.

A role as phagocytic receptor has also been suggested for the MR; however, this has been questioned e.g., by the finding that MR expression did not affect the uptake of *C. albicans* and zymosan (33, 41). In contrast, Rajaran et al. demonstrated that macrophages endocytose *M. tuberculosis* through MR by a mechanism that limits phago-lysosome fusion (42). We did not find any effect of *L. acidophilus* or *S. aureus* on the expression of MR, indicating that the bacteria do not employ the MR as a phagocytotic receptor. This is further supported by the increased killing and IL-12 production after mannan pre-stimulation as phago-lysosome fusion is a prerequisite for these effects. Thus, a direct employment of MR in endocytosis may involve another mechanism that the mechanism employed when the MR receptor is involved through a pre-stimulation with mannan. In contrast to the endocytosis of *L. acidophilus*, there was a direct effect of mannan on the endocytosis of FITC-dextran, known to bind and become endocytosed by the MR (25), thus demonstrating that the MR is involved in ligation and endocytosis of mannan. The clear inhibiting effect of CPZ on the mannan induced enhancement of IL-12 production but not on the IL-12 induced by microbial stimulation, which supports that the microbial induction of IL-12 and the MR induced enhancement are caused by two at least independent membrane events. The IL-12 enhancing effect of mannan but not the IL-12 induced by *L. acidophilus* was further abrogated by the inhibitor of clathrin mediated endocytosis, MDC, further supports this. The mannose receptor only has a short cytoplasmatic domain without any known signaling motif identified (43) and may thus not on its own be able to initiate a signaling pathway. Heinsbroek et al. found that MR was absent from the phagocytic cup upon uptake of *C. albicans* and zymosan but was transiently recruited to the phagosome at a later stage where it shortly after disappeared (33). This is in accordance with our finding that the effect of mannan on endosomal killing of the bacteria and on the *Ifnb* expression were transient, only appearing around 2–4 h after bacterial stimulation.

As pre-stimulation with mannan led to enhanced endocytosis of *L. acidophilus*, our finding supports that even though MR is not directly involved in forming the phagocytotic cup, it indirectly affects the bacterial endocytosis. Avota et al. demonstrated that DC-SIGN ligation led to increased sphingomyelinase activation (30). We did not measure the ASMase activity and hence, cannot firmly conclude that the same took place in our BMDCs. However, adding ASMase to the cells induced the same upregulation of IL-12 as the addition of mannan did. Moreover, nystatin that binds to cholesterol in the membranes inhibited the IL-12 production, but only after pretreatment with mannan. Together, this points to a shift toward more phagocytosis as opposed to macropinocytosis when BMDCs are pretreated with mannan. In accordance with Avota et al., we found upregulation of SLAMF1, which Avota et al. showed were co-expressed with ASMase upon DC-SIGN activation (28). Like MR, DC-SIGN is a lectin receptor with an internalization motif but without a signaling motif in its cytosolic part. While the DC-SIGN recruitment of SLAMF1 was essential for the enhanced endocytosis of measles virus into dendritic cells (28), our results do not allow any conclusion regarding a role of the increased plasma membrane expression SLAMF1 upon MR activation. Nevertheless, MR employment in BMDCs stimulated by Gram-positive bacteria induced increase in ROS and bacterial killing, a SLAMF1 dependent effect seen after bacterial stimulation (41).

In summary, we demonstrated here that mannan enhances the IL-12 production in BMDCs induced by some Gram-positive bacteria by enhancing endocytosis as well as endosomal killing and degradation of the bacteria, while the induction of IL-12 by *E. coli* is decreased by the presence of mannan. As many glycoproteins contain mannose residues, the response induced against e.g., allergens or vaccine antigens may thus depend on the microbial environment.

## Data Availability Statement

The datasets generated for this study are available on request to the corresponding author.

## Author Contributions

HF and RM: study concept, design, analysis, interpretation of data, and drafting of the manuscript. RM, HE, JS, EF, LL, VT, and HF: performance of experiments. All authors critically revised and approved the manuscript.

### Conflict of Interest

The authors declare that the research was conducted in the absence of any commercial or financial relationships that could be construed as a potential conflict of interest.
